# Minimally Invasive Surgery for Perihilar Cholangiocarcinoma: A Systematic Review of the Short- and Long-Term Results

**DOI:** 10.3390/cancers15113048

**Published:** 2023-06-03

**Authors:** Giammauro Berardi, Alessio Lucarini, Marco Colasanti, Germano Mariano, Stefano Ferretti, Roberto Luca Meniconi, Nicola Guglielmo, Marco Angrisani, Sofia Usai, Maria Carola Borcea, Giulia Canali, Giovanni Moschetta, Giuseppe Maria Ettorre

**Affiliations:** Department of General and Hepatobiliary and Pancreatic Surgery, Liver Transplantation Service, San Camillo-Forlanini Hospital, 00152 Rome, Italy; alessio.lucarini@uniroma1.it (A.L.);

**Keywords:** perihilar cholangiocarcinoma, minimally invasive surgery, Klatskin tumor, liliary tree cancer, cholangiocarcinoma, liver

## Abstract

**Simple Summary:**

The role of minimally invasive surgery (MIS) for perihilar cholangiocarcinoma (PHC) is still controversial. In this systematic review, we sought to investigate the safety and the postoperative and long-term outcomes of this technique. MIS for PHC is feasible, with safe postoperative and oncological outcomes. Further studies are needed to confirm these results, especially in the long term, since the evidence is still limited.

**Abstract:**

Surgery and postoperative systemic chemotherapy represent the standard treatment for patients with perihilar cholangiocarcinoma (PHC). Minimally Invasive Surgery (MIS) for hepatobiliary procedures has spread worldwide in the last two decades. Since resections for PHC are technically demanding, the role of MIS in this field is yet to be established. This study aimed to systematically review the existing literature on MIS for PHC, to evaluate its safety and its surgical and oncological outcomes. A systematic literature review on PubMed and SCOPUS was performed according to the PRISMA guidelines. Overall, a total of 18 studies reporting 372 MIS procedures for PHC were included in our analysis. A progressive increase in the available literature was observed over the years. A total of 310 laparoscopic and 62 robotic resections were performed. A pooled analysis showed an operative time ranging from 205.3 ± 23.9 and 840 (770–890) minutes, and intraoperative bleeding between 101.1 ± 13.6 and 1360 ± 809 mL. Minor and major morbidity rates were 43.9% and 12.7%, respectively, with a 5.6% mortality rate. R0 resections were achieved in 80.6% of patients and the number of retrieved lymph nodes ranged between 4 (3–12) and 12 (8–16). This systematic review shows that MIS for PHC is feasible, with safe postoperative and oncological outcomes. Recent data has shown encouraging results and more reports are being published. Future studies should address differences between robotic and laparoscopic approaches. Given the management and technical challenges, MIS for PHC should be performed by experienced surgeons, in high-volume centers, on selected patients.

## 1. Introduction

Cholangiocarcinomas (CCAs) are rare and heterogeneous tumors arising from the epithelial cells of the biliary tract. These malignancies represent approximately 15% of all primary liver neoplasms causing approximately 2% of all cancer-related deaths worldwide [1]. CCAs can be divided into three subtypes according to the anatomic location along the biliary tree: intrahepatic CCA (iCCA), perihilar CCA (PHC), and distal CCA (dCCA) [2]. As described by Klatskin in 1965, PHCs (also known as Klatskin tumors) represent half of the total number of CCAs diagnosed each year and arise between the second-order bile ducts and the insertion of the cystic duct onto the common bile duct (CBD) [3,4,5]. The most common symptom in patients presenting with PHC is painless jaundice. Given the sneaky presentation and the aggressive tumor biology, most patients are diagnosed with locally advanced or metastatic disease, thereby bearing a poor prognosis [5,6,7]. Multidisciplinary management of patients affected by PHC is key. Patients with early-stage (≤3 cm) unresectable pCCA, as well as individuals with disease arising in the setting of primary sclerosing cholangitis, may be candidates for liver transplantation [8,9]. Unresectable or metastatic patients are referred to systemic treatment and immunotherapy. Twenty-five percent of patients present with a disease that is amenable to resection [1]. In the era of multimodal treatment, the mainstay to achieve the best long-term survival is an R0 surgical resection followed by systemic chemotherapy [10]. Surgery for PHC is considered highly demanding due to the complexity of patients, the need for extended hepatectomies with bile duct reconstructions, and the proximity of the tumor to the hilar structures, namely the portal vein and the hepatic artery. Mortality in major series has been reported to be as high as 18%, while major morbidity (Clavien-Dindo ≥ 3) ranges between 27% and 54% [7,11,12,13].

In the last 20 years, the benefits of minimally invasive techniques have been proven in almost every field of abdominal surgical oncology [14,15,16,17,18]. Furthermore, despite the initial skepticism, laparoscopic and robotic-assisted techniques have been increasingly implemented in liver surgery over the last decade, without compromising the oncological outcomes [19,20,21,22,23]. However, the above-mentioned technical challenges and the fear of oncological inadequateness have limited the use of minimally invasive surgery (MIS) for PHC resections [24]. In 2010, Giulianotti et al. reported the first case of minimally invasive resection of a PHC using a robotic platform [25]. Since then, centers all over the world reported small series, including both laparoscopic and robotic-assisted cases. This paper aims to review all the available literature and summarize the evidence on the application of MIS for PHC resections. 

## 2. Materials and Methods

### 2.1. Literature Search

Preferred Reporting Items for Systematic Reviews and Meta-Analyses (PRISMA) statement guidelines for conducting and reporting systematic reviews were followed [26]. The research protocol was registered at the International Prospective Register of Systematic Reviews (http://www.crd.york.ac.uk/PROSPERO, accessed on 11 November 2022) with the following registration number: CRD42022375279. A systematic literature search was performed independently by two of the authors (A.L. and G.B.) through PubMed and Scopus. The search was limited to studies in humans and those reported in the English language. The period included in our search was from 1 January 2000 to 11 November 2022. The search strategy was based on different combinations of words for each database. For the PubMed database, the following combination was used: (“Laparoscopy”[Mesh] OR laparosc* [tiab] OR “Robotic Surgical Procedures”[Mesh] OR robot* [tiab] OR “Minimally Invasive Surgical Procedures”[Mesh] OR Minimally Invasive OR hybrid [tiab]) AND (“Cholangiocarcinoma”[Mesh] OR cholangiocarcinoma* [tiab] OR Klatskin[tiab] OR “Bile Duct Neoplasms”[Mesh] OR Bile Duct cancer*[tiab] OR Bile Duct neoplasm*[tiab]) OR perihil* [tiab] OR hilar [tiab] AND surg* [tiab]. The same keywords were inserted into the search manager fields of Scopus. Extensive cross-checking of the reference lists of all retrieved articles that fulfilled the inclusion criteria further broadened the search.

### 2.2. Study Selection

The same two authors independently screened the titles and abstracts of the studies that were identified in the database search through the online data extraction tool Covidence^®^ (Melbourne, Australia) (https://www.covidence.org/, accessed on 11 November 2022). Duplicate studies were automatically excluded. The following criteria were set for inclusion:

1. Studies reporting minimally invasive resections for PHCs. Robotic, laparoscopic, single-site, hand-assisted, or hybrid techniques were considered minimally invasive and included in the study.
2. Studies reporting at least one short-term and/or long-term outcome.
3. If more than one study was reported by the same institution, only the highest quality study was included.

The following exclusion criteria were set:

1. Original studies not reporting outcomes of patients undergoing MIS for PHC.
2. Review articles, letters, comments, and case reports.
3. Studies from which it was impossible to retrieve or calculate data of interest.

### 2.3. Data Extraction

The first reviewer (A.L.) extracted the data as follows: first author, year of publication, type of study, and country of origin. The two reviewers (A.L. and G.B.) then proceeded with data extraction on Covidence^®^ retrieving data as follows: study design, period of study, number of participants, number of patients undergoing MIS, age, gender, ASA score, body mass index (BMI), Bismuth-Corlette classification (I/II/IIIa/IIIb/IV), preoperative jaundice, operative time, blood loss, length of stay, conversion to laparotomy, negative margins (R0), type of hepatectomy, caudate lobectomy, number of lymph nodes retrieved, number of positive lymph nodes (N+), vascular resections, postoperative minor and major morbidity according to the Clavien-Dindo classification [27], mortality, duration of follow-up, recurrence rate, overall and disease-free survival rates. All relevant tables and figures were reviewed for data extraction. Whenever discrepancies between the two reviewers were found, a third reviewer (G.M.E) was consulted.

### 2.4. Risk of Bias

The Newcastle–Ottawa scale was used independently by the two authors to assess the quality of case-control studies.

## 3. Results

**Figure 1:**
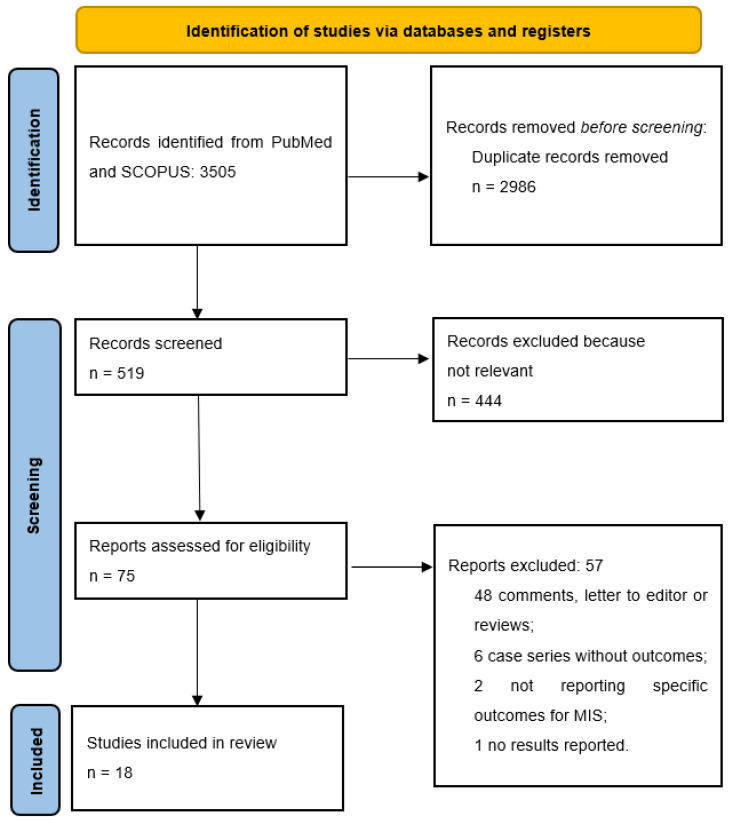
PRISMA flow diagram.

**Figure 2:**
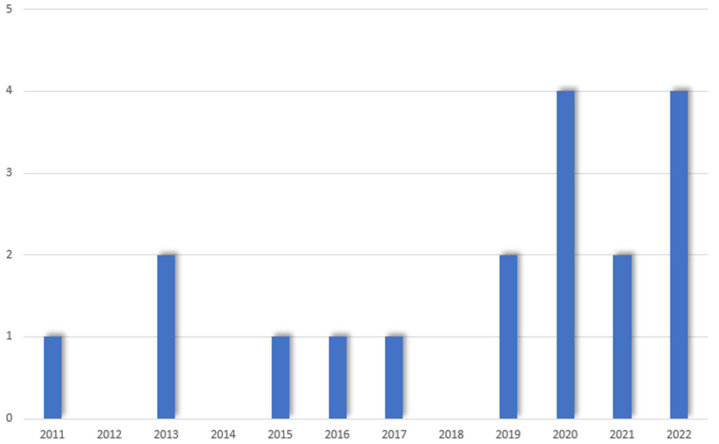
Papers published over the years.

The literature search yielded 1630 papers on PubMed and 1875 papers on Scopus. After duplicate removal, a total of 519 papers were screened. After title and abstract screening, 444 papers were deemed not relevant to the purpose of the study. A total of 75 papers underwent a full-text screening, and 57 were excluded for the following reasons: 48 papers were comments, letters to the editor or reviews, 6 papers were case series with no outcomes reported, 2 papers did not report specific outcomes of MIS, and 1 study did mention PHC without reporting any results (see Figure 1). Finally, a total of 18 studies reporting the outcomes of 372 patients were included in this review. There were no multiple studies from one institution. The articles were dated between January 2000 and March 2022. Over the years, an increasing trend in the number of publications was noted, with 12 out of 18 studies published after 2019 (see Figure 2). The vast majority of the studies (78%) were conducted in East Asia (13 from China [28,29,30,31,32,33,34,35,36,37,38,39,40], 1 in South Korea [41]), 2 in Italy [42,43], 1 in France [44] and 1 in the United States [45]. The articles consisted of 7 case-control studies and 11 case series; 15 studies reported on 310 patients undergoing laparoscopic procedures while 3 focused on 62 patients undergoing robotic surgery (see Table 1). Overall, 213/353 men (60.4%) and 140/353 (39.6%) women with an age ranging between 54 (36–77) and 73 (66–79) were included. According to the Bismuth-Corlette classification for PHC, 59/361 (16.3%) were type I, 94/361 (26%) were type II, 57/361 (15.8%) type IIIa, 95/361 (26.4%) type IIIb and 56/361 (15.5%) type IV [43]. Preoperative jaundice was diagnosed in 167/234 (71.4%) patients. Preoperative drainage was performed in 122/239 patients (51%) (see Table 2). The operative time ranged between 205.3 ± 23.9 and 840 (770–890) minutes, with a blood loss ranging between 101.1 ± 13.6 and 1360 ± 809 mL (see Table 3). A total of 33/328 (10%) patients were converted to open. Sixty-two out of 260 (23.8%) right hepatectomies and 124/260 (47.7%) left hepatectomies were performed. Caudate lobectomy was associated in 275/331 (83%) cases and vascular resections were required in 22/275 (8%) patients. The postoperative length of stay ranged between 5.9 ± 2.1 and 36.2 ± 9.5 days. An R0 resection was achieved in 271/336 (80.6%) patients. The number of retrieved lymph nodes ranged between 4 (3–12) and 12 (8–16) with a lymph node positivity rate of 29.3%. One hundred and six patients out of 241 (43.9%) developed minor postoperative morbidity and 44/344 (12.7%) developed major complications. A total of 44/358 (12.2%) bile leaks were reported. Twenty-one patients (5.6%) died postoperatively (see Table 4).

### 3.1. Laparoscopic Surgery

Fifteen studies focused on the laparoscopic approach for the resection of PHC, 9 case series, and 6 case-control studies (laparoscopy vs. open). A total of 310 patients were included in the analysis, 60.5% male and 39.5% female, with an age ranging between 53 (35–75) and 73 (66–79). The median BMI ranged between 22.2 (18–25.2) and 25 (21.2–28.7). According to the Bismuth-Corlette classification, 39 (12.5%) patients were diagnosed with type I, 87 (28%) with a type II, 48 (15.4%) with a type IIIa, 73 (23.5%) with a type IIIb and 52 (16.7%) with a type IV PHC. Preoperative jaundice was reported in 136/172 (79%) patients and drainage was performed in 92/239 (38.4%). The preoperative characteristics of the included studies are summarized in Table 2. The operative time ranged between 205.3 ± 23.9 and 610 (410–665) minutes, with a blood loss ranging between 101.1 ± 13.6 and 1360 ± 809 mL. A total of 32/276 (11.6%) patients were converted to open. Fifty-six out of 246 (22.8%) right hepatectomies and 116/246 (47.1%) left hepatectomies were performed, and caudate lobectomy was associated in 217/269 (80.7%) cases. A vascular resection was needed in 22/223 (9.9%) cases (Table 3). The postoperative length of stay ranged between 5.9 ± 2.1 and 36.2 ± 9.5 days. A negative margin (R0) was achieved in 226/274 (82.4%) patients. The retrieved lymph nodes number ranged between 4 (3–12) and 12 (8–16) and lymph node positivity was found in 59/194 (30.4%) patients. Seventy-four out of 179 patients (41.3%) developed minor morbidity and 36/282 (12.7%) experienced major complications. Thirty-seven out of 296 (12.5%) bile leaks were reported. Twenty patients (6.4%) died postoperatively (Table 4). Eight studies reported on the long-term oncological outcomes, while 4 [28,32,34,41] reported only the median follow-up with no oncological data. Three studies [30,35,38] reported the 1- and 2-years overall survival rate, ranging respectively between 62.5%–91.6% and 25%–52.1%. Only one study [32] reported a 3-year overall survival rate of 49.1% and a relapse-free survival rate of 47%.

### 3.2. Robotic Surgery

Three studies focused on the robotic-assisted approach for the resection of PHC, 2 case series, and 1 case-control study (robotic vs. open). A total of 62 patients were included in the analysis, 37 (59.7%) male and 25 (40.3%) female with an age ranging between 54 (36–77) and 62.4 ± 9. Only one study reported on the BMI, which was 23.7 ± 3.1. According to the Bismuth-Corlette classification, 20 (32.3%) were type I tumors, 7 (11.3%) were type II, 9 (14.5%) type IIIa, 22 (35.5%) type IIIb and 4 (6.4%) type IV PHC. Preoperative jaundice was reported in 46 (74.2%) patients. Preoperative drainage was performed in 30 (48.4%) patients (Table 2). Operative time ranged between 276 (170–500) and 840 (770–890) minutes, with blood loss ranging between 150 (20–1500) and 1360 ± 809 mL. Only 1/52 (1.9%) conversion to open was reported [42]. Caudate lobectomy was performed in 58 (93.5%) cases and no vascular resections were reported. A total of 6/14 (42.9%) right hepatectomies and 8/14 (57.1%) left hepatectomies were reported (Table 3). The postoperative length of stay ranged between 9 (4–52) and 16 (9–58) days. A negative margin (R0) was achieved in 45 (72.6%) patients. None of the three studies on robotic PHC resections reported on the number of lymph nodes retrieved, only on lymph node positivity which was found in 16 (25.8%) patients. Overall morbidity was 64.5% of which 32 (51.6%) were minor complications and 8 (12.9%) were major. Seven (11.3%) bile leaks were reported. Only 1 (1.6%) patient died postoperatively (Table 4). Two out of three studies on robotic surgery reported on the long-term oncological outcomes: Xu et al. [39] reported a median disease-free survival of 15.5 months (6–60), while Cillo et al. [42] reported a 75% disease-free survival and a 100% overall survival with a mean follow-up of 8.45 months.

## 4. Discussion

In this systematic review, we investigated the role of minimally invasive surgery in the treatment of patients affected by perihilar cholangiocarcinoma. The first result that can be drawn from our analysis is that the evidence on the topic is still limited, with few papers available to date, mainly retrospective case series or comparative studies that include few patients. Indeed, a total of 372 cases over 22 years was gathered from our search. Excluding the pioneer and experimental reports of the first cases back in early 2000, most of the centers recruited one or two cases per year. This is the result of the extreme selection process, which allows for improvement of the outcomes, or at least to keep them safe. This selection, however, comes with a long and steep learning curve which is difficult to overcome with few cases per year. Indeed, MIS in the setting of PHC is difficult to apply, since these patients often present with comorbidities, sarcopenia, jaundice requiring invasive interventions, infections, and sometimes advanced disease. For these reasons, and for the uncertainty of obtaining an adequate oncological resection, patients with PHC are frequently operated by standard open technique. Notwithstanding, some individuals present with a good preoperative condition, and favorable disease and anatomy. These patients seem to be good candidates for a minimally invasive approach, pushing on the advantages of MIS, without jeopardizing the oncological principles. Indeed, as a reflection of the selection process, patients included in our review were relatively young and fit. In these selected patients, MIS was associated with safe postoperative results, both in laparoscopy and in robotics.

Initial skepticism was triggered by the very first comparative study published back in 2016 by Xu et al. [39]. Indeed, the authors themselves stated that “results do not support continued practice of robotic surgery for PHC until significant technical and instrumental refinements are demonstrated”. Three years later, similar conclusions were drawn in the laparoscopic setting by Zhang et al. [38], showing no advantages compared to the open approach. However, in 2020 a propensity score-matched analysis by Ratti et al. [43] reported the first evidence of comparability between open and MIS. The authors indeed demonstrated similar outcomes between open and laparoscopic surgery in a well-conducted study. Subsequently, as of 2022, all the studies currently published disclosed the safety and feasibility of the minimally invasive approach, with reduced postoperative stay and complications compared to open [30,35,36,40]. Despite this, most of the papers give a word of caution on the steepness of the learning curve, the technical difficulties, and the high risks of potentially unexpected events, referring these procedures to experienced high-volume hepatobiliary surgeons working in specialized centers.

According to our review, conversion to open was 11.6% and 1.6% for laparoscopic and robotic surgery, respectively. While these two results cannot be compared, the higher rates of conversion in laparoscopy might be related to the technical challenges, especially during bile duct reconstructions. Indeed, one of the main advantages of the robotic platform is the easier suturing capacity enabled by the range of motion of the instruments, which cannot be reproduced in laparoscopy. This finding has yet to be confirmed by the literature, but it is worth mentioning and further investigating. Mortality and morbidity rates were low both in laparoscopy and robotic, and even lower compared to the 5%–18% mortality range reported in the literature for PHC resections [46]. This reflects the careful selection of candidates and the overall safety of the procedure in terms of postoperative results.

Concerning the oncological outcomes, unfortunately, only half of the manuscripts included in this review reported long-term survivals, with different follow-ups and non-comparable outcomes across studies. Overall and disease-free survival in these few studies was good, but further literature is needed to confirm the oncological safety of the procedure. As a surrogate of the oncological adequacy, the R0 resection rate reported was 82.4% for laparoscopy and 72.6% for robotics which is comparable to most of the series available to date. Another important surrogate of the oncological outcomes is the number of lymph nodes retrieved, which ranged from 4 to 12. This number seems comparable to the reported number of nodes retrieved in larger series in open surgery [47]. Furthermore, studies have demonstrated that the minimum number of lymph nodes to be retrieved in PHC resection should be four to allow proper staging [48]. With the above-mentioned oncological data available to date, we, unfortunately, cannot conclude on the long-term survivals of MIS for PHC, but we can suggest that it seems to be associated with oncological radicality and accurate staging and that further studies should disclose if this radicality and staging eventually translate into safe long-term survivals. A similar review reporting the outcomes of MIS for PHC was published in 2021 [49]. However, the search process of this well-conducted review was limited to July 2020, therefore, not capturing the six most recent and mature publications on the topic, which allowed us to double the number of patients included in the analysis.

This review has some limitations. First and foremost, the low quality of the studies, all retrospective and only a few being comparative or case-controlled, with few patients included. This is particularly true in the setting of robotics, which to date counts on three papers published, including a total of 62 patients. Despite the growing interest in the robotic platform in the setting of PHC, conclusions from our review should be interpreted with caution. Many of the papers included lacked data, such as inclusion or exclusion criteria for laparoscopy, the extent of lymphadenectomy, and the consequent number of yielded lymph nodes. Finally, almost 80% of the articles were from China, showing—as in gastric cancer [12,50,51,52]—an eastern–western effect in the treatment of PHC. Eastern patients have different characteristics as compared to the Western population (tumor presentation, BMI, etc.). Furthermore, perioperative management (drainage, stenting, embolization, etc.) and surgical techniques (lymphadenectomy, major hepatectomy, etc.) differ between the two countries. Therefore, the results of our study should be interpreted keeping in mind that most of the patients were treated in Eastern centers [16,51,52].

## 5. Conclusions

Regardless of the surgical technique, PHC represents a challenging disease to manage. As recommended by all guidelines, patients affected by PHC should be managed in a multidisciplinary setting in the hands of an expert physician [53]. A high experience in both open procedures for PHC and MIS of the liver is key. Minimally invasive surgery for patients with perihilar cholangiocarcinoma is feasible, with good perioperative outcomes and safe oncological results, both with the laparoscopic and robotic approaches. Data are still immature to disclose the superiority of one or the other technique, or to compare its results with the open approach. MIS for PHC must be performed in high-volume hepatobiliary units in the hands of expert minimally invasive surgeons, to ensure safe postoperative outcomes, adequate oncological radicality, and to recognize and treat potential unexpected events accordingly.

## Figures and Tables

**Table 1:** Characteristics of the included studies.

First Author	Country	Year	Study Design	Technique	Period of Study	Total No. of Patients	NOS Scale
Elmoghazy et al. [44]	France	2019	Case series	Laparoscopy	January 2002–January 2016	11	NA
Feng et al. [29]	China	2019	Case series	Laparoscopy	November 2016–November 2018	9	NA
Ratti et al. [43]	Italy	2020	Case-control (PSM)	Laparoscopyvs. open	January 2014–June 2019	48	8/8
Gumbs et al. [45]	USA	2013	Case series	Laparoscopy	12/2002–June 2011	5	NA
Yu et al. [37]	China	2011	Case series	Laparoscopy	September 2006–December 2008	14	NA
Chen et al. [28]	China	2013	Case series	Laparoscopy	January 2000–November 2011	36	NA
Ma et al. [40]	China	2022	Case-control (PSM)	Laparoscopyvs. open	February 2017–August 2020	149	8/8
Zhang et al. [38]	China	2020	Case-control	Laparoscopyvs. open	January 2015–December 2018	23	7/8
Qin et al. [35]	China	2022	Case-control (PSM)	Laparoscopyvs. open	January 2013–October 2018	166	8/8
Xiong et al. [36]	China	2022	Case-control	Laparoscopyvs. open	January 2018–January 2020	64	7/8
Li et al. [33]	China	2017	Case series	Laparoscopy	October 2007–May 2014	9	NA
Lee et al. [41]	South Korea	2015	Case series	Laparoscopy	August 2014–December 2014	5	NA
He et al. [30]	China	2022	Case-control (PSM)	Laparoscopyvs. open	January 2018–March 2022	21	8/8
Liu et al. [34]	China	2020	Case series	Laparoscopy	April 2015–October 2018	6	NA
Li et al. [32]	China	2021	Case series	Laparoscopy	December 2015–November 2019	32	NA
Li et al. [31]	China	2020	Case series	Robotic	March 2017–February 2019	48	NA
Xu et al. [39]	China	2016	Case-control	Robotic vs. open	May 2009–October 2012	42	6/8
Cillo et al. [42]	Italy	2021	Case series	Robotic	March 2019–March 2020	4	NA

PSM: Propensity Score Matching, NA: Not Applicable.

**Table 2:** Preoperative characteristics.

First Author	No of MIS	Age	Sex (M/F)	BMI	Bismuth-Corlette					Preoperative Jaundice	Preoperative Drainage
					I	II	IIIa	IIIb	IV		
Elmoghazy et al. [44]	11	62 (55–79)	8/3	24 (21–33)	ND					3 (27%)	3 (27%)
Feng et al. [29]	9	62 ± 4.92	5/4	22.98 ± 4	0	0	2	5	2	4 (44%)	4 (44%)
Ratti et al. [43]	16	61 (48–81)	8/8	25 (21.2–28.7)	1	5	5	5	0	14 (87%)	12 (75%)
Gumbs et al. [45]	5	73 (66–79)	ND	ND	0	0	3	2	0	ND	ND
Yu et al. [37]	14	55.7 (51–71)	ND	ND	8	6	0	0	0	11 (78%)	0
Chen et al. [28]	36	66 ± 7.87	27/9	ND	17	19	0	0	0	ND	ND
Ma et al. [40]	20	61.9 ± 9	16/4	22.2 ± 2.8	2	4	2	7	5	14 (70%)	12 (60%)
Zhang et al. [38]	14	65.4 ± 8.9	7/7	23.1 ± 3.1	5	0	8	1	0	ND	7 (50%)
Qin et al. [35]	83	62.06 ± 9.44	44/39	22.65 ± 3.11	2	41	5	11	24	ND	ND
Xiong et al. [36]	34	55 (39–64)	18/16	22.2 (18–25.2)	2	2	10	17	3	22 (65%)	22 (65%)
Li et al. [33]	9	62.7 (50–74)	6/3	ND	1	3	0	4	1	9 (100%)	ND
Lee et al. [41]	5	63 (43–76)	5/0	ND	1	1	1	2	0	5 (100%)	4 (80%)
He et al. [30]	16	64 (54–66) *	7/9	23.5 ± 2.45	0	0	7	9	0	15 (94%)	12 (75%)
Liu et al. [34]	6	53 (35–75)	4/2	ND	0	0	1	2	3	2 (33%)	2 (33%)
Li et al. [32]	32	60.6 (39–77)	21/11	<18.5: 1 18.5–24.9: 23 25–29.9: 8	0	6	4	8	14	22 (69%)	14 (44%)
Li et al. [31]	48	62.4 ± 9	28/20	23.7 ± 3.1	20	6	5	17	0	33 (69%)	20 (42%)
Xu et al. [39]	10	54 (36–77)	8/2	ND	0	1	4	1	4	10 (100%)	6 (60%)
Cillo et al. [42]	4	60.5 (44–79)	1/3	ND	0	0	0	4	0	3 (75%)	4 (100%)

Data reported as mean ± standard deviation or median (range) as appropriate. *: Interquartile Range (IQR). MIS: Minimally Invasive Surgery. BMI: Body Mass Index. ND: Not Determined.

**Table 3:** Operative data.

First Author	Operative Time (min)	Blood Loss (mL)	Conversion to Open	Type of Liver Resection	Caudate Resection	Vascular Resection	Blood Transfusion
Elmoghazy et al. [44]	355 (290–420)	250 (120–1200)	5 (45%)	8 MajH, 3 ER	6 (55%)	6 (55%)	3
Feng et al. [29]	479.6 ± 98.1	950 ± 800	0	4 RH, 5 LH	9 (100%)	0	4
Ratti et al. [43]	360 ± 290	380 ± 250	3 (18%)	7 RH, 9 LH	16 (100%)	3 PV, 3 HA	2
Gumbs et al. [45]	ND	240 (0–400)	1 (20%)	3 RH, 2 LH	ND	0	ND
Yu et al. [37]	305(210–445)	386 (200–1000)	0	ND	5 (36%)	0	0
Chen et al. [28]	205.3 ± 23.9	101.1 ± 13.6	0	ND	ND	ND	ND
Ma et al. [40]	307.8 ± 90.8	1360 ± 809	7 (35%)	2 RH, 8 LH, 1 MinH	7 (35%)	3 (15%)	7
Zhang et al. [38]	519.4 ± 115.4	821.4 ± 713.8	2 (14%)	ND	14 (100%)	ND	6
Qin et al. [35]	360 (300–420)	300 (100–500) *	9 (11%)	8 RH, 41 LH, 1 RS, 1 LS	83 (100%)	1 PV, 3 HA	21
Xiong et al. [36]	475.5 (219–630)	300 (50–3500)	ND	10 RH, 22 LH, 2 CH	34 (100%)	1 (3%)	ND
Li et al. [33]	450 (330–540)	503 (150–850)	0	2 LH	4 (44%)	0	ND
Lee et al. [41]	610 (410–665)	650 (450–1300)	0	1 RH, 2 LH, 2 BDR	3 (60%)	ND	ND
He et al. [30]	489.6 ± 79.1	300 (200–400) *	0	7 RH, 9 LH	16 (100%)	1 PV (6%)	8
Liu et al. [34]	590 (540–660)	400 (300–500)	0	1 RH, 5 LH	6 (100%)	1 (17%)	0
Li et al. [32]	476.9 ± 133.8	568.7 ± 324	5 (16%)	13 RH, 11 LH	14 (44%)	ND	2.5 **
Li et al. [31]	276 (170–500)	150 (20–1500)	0	ND	48 (100%)	0	13
Xu et al. [39]	703 ± 62	1360 ± 809	ND	6 RH, 4 LH	10 (100%)	ND	6
Cillo et al. [42]	840 (770–890)	700 (600–800)	1 (25%)	4 LH	0	0	0

Data reported as mean ± standard deviation or median (range) as appropriate. *: Interquartile Range (IQR), **: Blood Units, ND: Not Determined, RH: Right Hepatectomy, LH: Left Hepatectomy, MajH: Major Hepatectomy, MinH: Minor Hepatectomy, ER: Extended Resection, CH: Central Hepatectomy, BDR: Bile Duct Resection, RS: Right Segmentectomy, LS: Left Segmentectomy, PV: Portal Vein, HA: Hepatic Artery.

**Table 4:** Postoperative data.

First Author	Length of Stay (Days)	R0 Resection	Lymph Node Retrieved	Positive Lymph Node	Morbidity (C.D.)		Bile Leak	Mortality
					I-II	III-IV		
Elmoghazy et al. [44]	21 (10–57)	8 (73%)	9 (0–19)	5 (45%)	4	3	4 (36%)	2 (18%)
Feng et al. [29]	36.2 ± 9.5	9 (100%)	ND	ND	1	1	1 (11%)	1 (11%)
Ratti et al. [43]	10 (7–15)	13 (81%)	12 (8–16)	6 (37%)	5	2	3 (18%)	0
Gumbs et al. [45]	15 (11–21)	4 (80%)	ND	ND	ND		ND	0
Yu et al. [37]	Type I: 9 (6–22)	10 (71%)	ND	ND	ND		0	5 (36%)
	Type II: 19 (9–25)							
Chen et al. [28]	5.9 ± 2.1	ND	ND	ND	1	0	1 (2.7%)	0
Ma et al. [40]	14.5 ± 5	14 (70%)	ND	4 (20%)	ND	4	1 (5%)	1 (5%)
Zhang et al. [38]	17.8 ± 7.1	7 (50%)	9.7 ± 6.7	ND	3	5	5 (36%)	1 (7%)
Qin et al. [35]	14 (10–19) *	78 (94%)	8 (5–10) *	17 (20%)	ND	10	8 (10%)	7 (8%)
Xiong et al. [36]	20 (10–44)	32 (94%)	9.50 (6–15)	11 (32%)	31	3	8 (23%)	0
Li et al. [33]	15.7 (10–27)	9 (100%)	ND	9 (100%)	ND		ND	2 (22%)
Lee et al. [41]	12 (9–21)	4 (80%)	4 (3–12)	0	0	1	1 (20%)	0
He et al. [30]	11.5 (10–17.7) *	15 (94%)	7 (5–8) *	7 (44%)	14	2	0	0
Liu et al. [34]	16 (13–24)	4 (66%)	ND	ND	0	1	1 (17%)	0
Li et al. [32]	23.3 ± 11.7	19 (59%)	8.93 ± 5.26	ND	15	4	4 (17%)	1 (3%)
Li et al. [31]	9 (4–52)	35 (73%)	ND	11 (23%)	23	5	2 (4%)	0
Xu et al. [39]	16 (9–58)	7 (70%)	ND	3 (30%)	6	3	4 (40%)	1 (10%)
Cillo et al. [42]	9 (7–11)	3 (75%)	ND	2 (50%)	3	0	1 (25%)	0

Data reported as mean ± standard deviation or median (range) as appropriate. *: Interquartile Range (IQR). ND: Not Determined.

## Data Availability

Data available on request of authors.
